# Prevalence and Causes of Diagnostic Errors in Hospitalized Patients Under Investigation for COVID-19

**DOI:** 10.1007/s11606-023-08176-6

**Published:** 2023-03-23

**Authors:** Andrew D. Auerbach, Gopi J. Astik, Kevin J. O’Leary, Peter N. Barish, Molly A. Kantor, Katie R. Raffel, Sumant R. Ranji, Stephanie K. Mueller, Sharran N. Burney, Janice Galinsky, Esteban F. Gershanik, Abhishek Goyal, Pooja R. Chitneni, Sarah Rastegar, Armond M. Esmaili, Cynthia Fenton, Anunta Virapongse, Li-Kheng Ngov, Marisha Burden, Angela Keniston, Hemali Patel, Ashwin B. Gupta, Jeff Rohde, Ruby Marr, S. Ryan Greysen, Michele Fang, Pranav Shah, Frances Mao, Farah Kaiksow, David Sterken, Justin J. Choi, Jigar Contractor, Abhishek Karwa, David Chia, Tiffany Lee, Colin C. Hubbard, Judith Maselli, Anuj K. Dalal, Jeffrey L. Schnipper

**Affiliations:** 1 Division of Hospital Medicine, Department of Medicine, University of California, San Francisco, San Francisco, CA USA; 2 Division of Hospital Medicine, Northwestern University Feinberg School of Medicine, Chicago, IL USA; 3 Division of Hospital Medicine, Department of Medicine, University of Colorado Anschutz Medical Campus, Aurora, CO USA; 4 Division of Hospital Medicine, Zuckerberg San Francisco General Hospital, San Francisco, CA USA; 5 Hospital Medicine Unit, Division of General Internal Medicine and Primary Care, Brigham and Women’s Hospital, and Harvard Medical School, Boston, MA USA; 6 Brigham and Women’s Faulkner Hospital, Boston, MA USA; 7 Division of Hospital Medicine, University of Michigan Medical School, Ann Arbor, MI USA; 8 Division of Hospital Medicine, VA Ann Arbor Healthcare System, Ann Arbor, MI USA; 9 Section of Hospital Medicine, Perelman School of Medicine, University of Pennsylvania, Philadelphia, PA USA; 10 Division of Hospital Medicine, University of Wisconsin School of Medicine and Public Health, WI Madison, USA; 11 Department of Medicine, Weill Cornell Medical College, New York, NY USA

## Abstract

**Background:**

The COVID-19 pandemic required clinicians to care for a disease with evolving characteristics while also adhering to care changes (e.g., physical distancing practices) that might lead to diagnostic errors (DEs).

**Objective:**

To determine the frequency of DEs and their causes among patients hospitalized under investigation (PUI) for COVID-19.

**Design:**

Retrospective cohort.

**Setting:**

Eight medical centers affiliated with the Hospital Medicine ReEngineering Network (HOMERuN).

**Target population:**

Adults hospitalized under investigation (PUI) for COVID-19 infection between February and July 2020.

**Measurements:**

We randomly selected up to 8 cases per site per month for review, with each case reviewed by two clinicians to determine whether a DE (defined as a missed or delayed diagnosis) occurred, and whether any diagnostic process faults took place. We used bivariable statistics to compare patients with and without DE and multivariable models to determine which process faults or patient factors were associated with DEs.

**Results:**

Two hundred and fifty-seven patient charts underwent review, of which 36 (14%) had a diagnostic error. Patients with and without DE were statistically similar in terms of socioeconomic factors, comorbidities, risk factors for COVID-19, and COVID-19 test turnaround time and eventual positivity. Most common diagnostic process faults contributing to DE were problems with clinical assessment, testing choices, history taking, and physical examination (all *p* < 0.01). Diagnostic process faults associated with policies and procedures related to COVID-19 were not associated with DE risk. Fourteen patients (35.9% of patients with errors and 5.4% overall) suffered harm or death due to diagnostic error.

**Limitations:**

Results are limited by available documentation and do not capture communication between providers and patients.

**Conclusion:**

Among PUI patients, DEs were common and not associated with pandemic-related care changes, suggesting the importance of more general diagnostic process gaps in error propagation.

## INTRODUCTION

Diagnostic errors (DEs) are “the failure to (a) establish an accurate and timely explanation of the patient’s health problem(s) or (b) communicate that explanation to the patient.”^1^ Many factors contribute to diagnostic errors, but key among them are complex and fragmented care systems, the limited time available to providers trying to ascertain a firm diagnosis, and the work systems and cultures that impede improvements in diagnostic performance.^2–8^ In the hospital setting, work burden, patient acuity, and technology (such as electronic health records [EHRs] and multiple “alerting” systems^9^) all contribute.

In the early stages of the COVID-19 pandemic, these preexisting problems were exacerbated in ways that have yet to be fully elucidated.^10^ Shortages of personal protective equipment (PPE) and concerns about workforce preservation led hospitals to replace physical visits with videoconferencing or telephone-based encounters.^11–17^ Hospital visitor restrictions impaired or delayed collaborative discussions with patients’ family members, potentially limiting clinicians’ ability to obtain thorough clinical histories. Changes in coverage models (e.g., internal medicine providers providing critical care services^18^) changed the clinical expertise of physicians caring for COVID-19 patients. Data from our network suggested that half of hospitalist leaders surveyed related a missed or delayed non-COVID-19 diagnosis among patients under investigation (PUI) for COVID-19 infection. A similar proportion also reported missing COVID-19 as a diagnosis in patients admitted for other medical reasons,^13^ consistent with conceptual models published early in the pandemic.^10^

This study, undertaken at the height of the first wave of the COVID-19 pandemic, sought to gain an understanding of the prevalence of diagnostic errors among PUIs or with confirmed COVID-19 infection and to gather insights into whether changes in health care policies and procedures during the pandemic might have contributed to these errors.

## METHODS

### Study Design

This was a retrospective multicenter cohort study of randomly selected patients admitted under investigation for COVID-19 investigation.

### Sites and Subjects

This study was undertaken as a collaboration among eight academic centers participating in the Hospital Medicine ReEngineering Network^19^ who were already conducting diagnostic error case reviews as part of a larger research study.^20^ Sites in this study represented a range of settings, including locations such as New York City which were affected more significantly by the pandemic than others during our study time period.

Patients for this study were admitted between February and July 2020 and identified through examination of infection control logs maintained at each site listing patients’ initial COVID-19 status. Patients were then considered for review if they had signs or symptoms considered high risk for COVID-19 based on Centers for Disease Control and Prevention definitions at the time of our study (for example, travel to a high-prevalence area, congregate-living settings, loss of smell) and were awaiting a COVID-19 test or had a negative test prior to hospitalization but persistent symptoms prompting an additional test. We excluded patients whose tests were obtained under universal screening programs (e.g., of all admitted patients regardless of signs or symptoms of COVID-19 infection). As we have done in previous studies,^21^ we employed a block randomization schema based on patient medical record numbers to randomly select patients; sites were asked to review a minimum of 4 and up to 8 cases meeting these criteria per site per month.

### Adjudicator Training

Chart reviewers were identified and trained as part of our larger study, which was ongoing at the start of the pandemic. Each reviewer was first trained to identify diagnostic errors by reviewing at least 5 “gold standard cases” with expert reviewers, then by carrying out adjudications of sample cases in pairs and groups from all participating centers to gain expertise and to cross check results within and across sites.

Group training was followed by review of 10 additional standard cases until we observed consistent agreement on diagnostic error determinations within and across sites, based on over-reads by coordinating center collaborators and through multisite webinar-based case reviews. Once consistency was achieved, we proceeded with adjudication of real cases, with each case being reviewed by two clinicians (physician, nurse practitioner, or physician assistant) active in inpatient care, each of whom was trained according to the protocol above. Finally, every 10^th^ real case was overread by expert reviewers to ensure consistency in error determinations across sites.

### Determination of Errors and Underlying Causes

Our two-reviewer process focused on data obtainable from charts, such as patient testing reasons, any COVID-19-specific symptoms which might modify diagnostic thinking (e.g., new-onset anosmia), and patient factors such as functional status.

Adjudicators examined, discussed, and entered data for each case jointly. As a result, each adjudication represented the shared viewpoint of two trained clinicians not connected to the case. In cases of disagreement, a third expert reviewer was employed to help reach a final determination.

Diagnostic errors, defined as missed or delayed diagnoses, were identified using the SAFER-Dx framework, modified for the inpatient setting as operationalized in a larger study (conducted by our group) of diagnostic errors in medical patients who died or were transferred to the ICU.^20^

Our review methodology used the framework of a “working diagnosis,” in which clinical thinking can rightly evolve over time and can be represented by patterns in diagnostic testing and empiric treatment. Reviewers examined the entire medical record, with particular focus on relating documentation to the results and timestamps for objective data such as vital sign records, laboratory tests, and orders. For example, if a diagnosis was not documented and did not lead to orders for its treatment (or for definitive diagnostic testing) until well after it was apparent based on laboratory findings, then this would be considered a diagnostic error.

If making a diagnosis required a timely procedure (such as urgent endoscopy for gastrointestinal bleeding), but that procedure could not be performed due to various system factors, we would have considered the event to represent a delay in diagnosis because an ideal health care system would be able to accommodate this procedure request. Finally, we granted some discretion to providers based on the context of the information available to clinicians at the time of documentation. This last standard was particularly applicable in our cases where COVID-19 infection was a consideration, as therapeutic and diagnostic approaches were rapidly evolving during our study time period.

Each case was also reviewed for diagnostic process faults using the Diagnostic Error Evaluation and Research (DEER) taxonomy^2,22,23^ framework, an approach useful for characterizing diagnostic processes regardless of whether or not a diagnostic error took place. DEER is composed of 9 major groupings, under which are more than 50 potential diagnostic process faults that represent various underlying causes of diagnostic errors. Because DEER has the greatest evidence for use in primary care settings and has not been applied to inpatient care, we expanded the taxonomy to include inpatient scenarios (such as transfers from outside hospitals); these factors were added to major headings and analyzed as additional factors.^24^ We also generated a set of diagnostic process faults related to COVID-19 care (Appendix Table 7). COVID-19-specific diagnostic process factors were generated based on expert input from our collaborative group and included faults such as “physical examination limitations due to medical distancing.” COVID-19-related diagnostic process faults were then aggregated into a separate grouping as a new predictor in analytic models (described below). Finally, each case with an error was rated in terms of its harm to the patient using the NCC-MERP harm rating scale.^25^

### Outcomes and Predictors

The primary outcome of this study was the presence or absence of a diagnostic error, defined as any missed or delayed diagnosis during the index hospitalization. Secondary analyses examined harms due to diagnostic errors. Key predictors tested in primary analyses were those with statistical association with DE in unadjusted analyses and included all major groupings of the DEER taxonomy, including COVID-19-related process faults.

### Analytic Approach

We first characterized patients with and without DE using bivariate statistics. Differences in characteristics between patients with and without DE were assessed using either the chi-square test or Fisher exact test for categorical variables and Wilcoxon rank sum test for continuous variables.

Prevalence ratios comparing patients with DE versus those without were computed from the logistic models, as were confounder-adjusted attributable fractions, i.e., the proportion of DE that would have been eliminated if that process fault were eliminated.^26,27^ Our primary analyses, based on our hypothesis that DEER process faults would be associated with diagnostic errors, used multivariable logistic regression models to assess the adjusted associations between DEER process fault categories with diagnostic errors. Because the DEER taxonomy major grouping for the “clinical assessment” fault was highly correlated with diagnostic error (*R*^2^ = 0.70, accuracy = 0.91, sensitivity = 0.89, specificity = 0.91), we carried out exploratory models examining other DEER factors associated with DE but excluding the Clinical Assessment Fault DEER taxonomy grouping. We tested for trends in error rates over the period of our study using the Cochrane-Armitage test for trend. Finally, we calculated descriptive statistics of NCC-MERP harm ratings; our sample size and error rate were too low to carry out multivariable models determining the association between DEER factors and harms due to diagnostic errors. All analyses were conducted using R Statistical Software (v4.1.2; R Core Team 2021).

## RESULTS

### Patient Characteristics and COVID-19 Illness Features

Two hundred and fifty-seven patients were randomly selected for inclusion in our study. Of these, 36 (14%) had a diagnostic error. Patients with and without diagnostic errors were statistically similar in terms of age, race, ethnicity, primary language, comorbidities, and functional and social determinants of health, but were noted to have symptoms of delirium or be socially isolated more often in chart notation (Table 1). Patients with and without DE were also similar in the proportion of clinical features that might have influenced clinical diagnosis of COVID-19 or non-COVID-19 reasons for hospitalization, including exposure history, travel history, presenting symptoms, severity of illness (e.g., need for mechanical ventilation), and COVID-19 test turnaround times (Table 2).

**Table 1:** Patient Characteristics (*n* = 257)

Characteristic	Diagnostic error present (*n* = 36, 14.0%)	No diagnostic error present (*n* = 221, 86.0%)	*p* value
Age			0.45
18–40	6 (16.7)	27 (12.2)	
41–50	3 (8.3)	30 (13.6)	
51–60	4 (11.1)	43 (19.5)	
61–70	6 (16.7)	46 (20.8)	
71–80	8 (22.2)	43 (19.5)	
> 80	9 (25.0)	32 (14.5)	
Gender			> 0.99
Male	19 (52.8)	115 (52.0)	
Female	17 (47.2)	105 (47.5)	
Other	0 (0)	1 (0.5)	
Race			0.71
Asian	2 (5.6)	12 (5.4)	
Black	6 (16.7)	39 (17.6)	
White	18 (50.0)	90 (40.7)	
Unknown	10 (27.8)	80 (36.2)	
Ethnicity			0.15
Hispanic	4 (11.1)	45 (20.4)	
Not Hispanic	28 (77.8)	133 (60.2)	
Unknown	4 (11.1)	43 (19.5)	
English is primary language			0.24
Yes	32 (88.9)	172 (77.8)	
No	3 (8.3)	42 (19.0)	
Unknown	1 (2.8)	7 (3.2)	
Comorbidities noted in chart			
Renal failure	10 (28.6)	52 (23.5)	0.23
Heart failure	8 (22.2)	31 (14.0)	0.31
Chronic lung disease	14 (38.9)	68 (30.9)	0.52
Insulin-dependent diabetes	6 (16.7)	32 (14.5)	0.24
Non-insulin-dependent diabetes	6 (16.7)	41 (18.7)	0.33
Coronary artery disease	5 (13.9)	38 (17.2)	0.76
Congestive heart failure	11 (30.6)	36 (16.3)	0.09
Chronic kidney disease on dialysis	4 (11.1)	10 (4.5)	0.16
Chronic kidney disease—no dialysis	4 (11.1)	36 (16.5)	0.55
Liver disease	1 (2.8)	19 (8.6)	0.54
Cancer	3 (8.3)	29 (13.1)	0.84
Current smoker	5 (13.9)	32 (14.5)	0.83
Former smoker	18 (50.0)	71 (32.1)	0.11
HIV	1 (2.9)	9 (4.1)	0.19
Chart notation of any of the following			
Housing instability	1 (2.8)	5 (2.3)	> 0.99
Unhoused	1 (2.8)	9 (4.1)	> 0.99
Difficulty paying bills	2 (5.6)	3 (1.4)	0.15
Lack of reliable transportation	1 (2.8)	4 (1.8)	0.53
Unemployment	2 (5.6)	5 (2.3)	0.25
Loneliness-isolation	4 (11.1)	4 (1.8)	0.02
Depression	4 (11.1)	19 (8.6)	0.54
Active mental illness	2 (5.6)	17 (7.7)	> 0.99
Alcohol > 5 drinks/day	3 (8.3)	13 (5.9)	0.48
Any tobacco use	4 (11.1)	32 (14.5)	0.80
Illicit drugs	2 (5.6)	15 (6.8)	> 0.99
Limitations in activities of daily living	5 (13.9)	40 (18.1)	0.64
Limitations in instrumental activities of daily living	5 (13.9)	45 (20.4)	0.50

**Table 2:** COVID-19-Specific Factors

Characteristic	Error present (*n* = 36, 14.0%)	No error present (*n* = 221, 86.0%)	*p* value
Patient test status at admission			
Test obtained at admission based on concern for COVID-19, with no recent testing	31 (86.1)	187 (85.0)	0.86
Test obtained at admission based on concern for COVID-19, with recent negative test	3 (8.3)	22 (10.0)	> 0.99
Test result positive prior to admission, but admitted for repeat testing due to symptoms	2 (5.6)	10 (4.5)	0.68
Test result positive during hospitalization	9 (25.0)	65 (30.0)	0.55
Exposures in previous 14 days			
Household-related COVID-19 contact	3 (8.3)	10 (4.5)	0.40
Community-related COVID-19 contact	1 (2.8)	11 (5.0)	> 0.99
Health care	0 (0)	4 (1.8)	> 0.99
Congregate-living contact with lab-confirmed COVID-19 case	0 (0)	3 (1.4)	> 0.99
Occupational/other	0 (0)	2 (0.9)	> 0.99
Patient was a health care worker	1 (2.9)	9 (4.2)	> 0.99
In past 14 days—any risk factors			
Congregate living	1 (2.8)	12 (5.4)	> 0.99
Skilled nursing facility	3 (8.3)	17 (7.7)	> 0.99
Unable to shelter in place	1 (2.8)	9 (4.1)	> 0.99
Working in essential occupation	1 (2.8)	8 (3.6)	> 0.99
Other	4 (11.1)	13 (5.9)	0.27
Clinical features			
Pneumonia	18 (50.0)	121 (55.0)	0.70
Acute respiratory distress syndrome	5 (13.9)	30 (13.6)	> 0.99
Abnormal chest radiograph	25 (69.4)	149 (68.0)	> 0.99
Admitted to ICU	12 (33.3)	54 (24.4)	0.26
Need for invasive mechanical ventilation	5 (41.7)	35 (64.8)	0.19
Deep venous thrombosis	2 (5.6)	11 (5.0)	0.61
Pulmonary embolism	2 (5.6)	10 (4.5)	0.84
Signs and symptoms at admission			
Fever > 38 °C	12 (33.3)	74 (33.5)	0.80
Subjective fevers	15 (41.7)	95 (43.2)	0.93
Chills	10 (27.8)	60 (27.3)	0.22
Myalgias	13 (36.1)	51 (23.2)	0.25
Rhinorrhea	1 (2.8)	15 (6.8)	0.84
Sore throat	3 (8.3)	21 (9.6)	0.83
Cough	15 (41.7)	121 (54.8)	0.19
Shortness of breath	18 (50.0)	136 (61.8)	0.37
Nausea/vomiting	9 (25.0)	53 (24.0)	0.55
Headache	4 (11.4)	36 (16.4)	0.74
Abdominal pain	8 (22.2)	32 (14.5)	0.46
Diarrhea	9 (25.0)	49 (22.2)	0.84
Loss of taste or smell	0 (0)	6 (2.7)	0.47
Delirium	2 (5.6)	44 (19.9)	0.03
Patient’s documentation included another possible diagnosis	33 (91.7)	176 (80.0)	0.09
Median minutes elapsed between the time COVID-19 test was ordered and final result returned (IQR)	200 (120.5, 554.5)	250.5 (110.5, 720)	0.48

### DEER Taxonomy Features Associated with Diagnostic Errors

Components of the DEER taxonomy features are listed in Appendix Table 7. In unadjusted analyses (Table 3), faults in history taking, physical exam, testing ordering/performance/interpretation, patient follow-up and monitoring, teamwork, and clinical assessment were significantly associated with diagnostic errors (all *p* < 0.01). DEER diagnostic process faults related to COVID-19 (e.g., failures or delays in eliciting a critical piece of history or physical exam finding or erroneous clinician interpretation of a test related to COVID-19, failure or delay in recognizing or acting upon urgent conditions or complications due to medical distancing) were significantly more frequent among patients with DE (29.5% vs. 9.2%, *p* < 0.01). In models adjusting for all potential diagnostic process faults (Table 4), only clinical assessment problems remained significantly associated with diagnostic errors, and with a high attributable fraction (78.79% of DEs potentially eliminated if clinical assessment problems eliminated, 95% CI 55.6–102.0). In models without clinical assessment as a covariate, only three non-COVID-19-related process faults had statistically significant attributable fractions (history taking, physical examination, and test ordering, performance, or interpretation), with estimated reductions in diagnostic error rates of between 20 and 37% if these process faults were eliminated entirely.

**Table 3:** Unadjusted Association Between Diagnostic Process Faults and Diagnostic Errors (*n* = 257)

	No. of errors/no. of cases (%)			
DEER diagnostic process fault^*^	Prevalence of error with fault	Prevalence of error with no fault	Unadjusted prevalence ratio (PR) (95% CI)	*p* value
Access and presentation	6/31 (19.4)	30/226 (13.3)	1.46 (0.5–2.9)	0.36
History taking	21/67 (31.3)	15/190 (7.9)	3.97 (2.3–6.9)	< 0.01
Physical exam	13/23 (56.5)	23/234 (9.8)	5.75 (3.4–10.1)	< 0.01
Testing ordering, performance, and interpretation	22/51 (43.1)	14/206 (6.8)	6.35 (3.3–13.4)	< 0.01
Patient follow-up and monitoring	12/21 (57.1)	24/236 (10.2)	5.62 (3.0–9.6)	< 0.01
Consultation and referral	6/25 (24.0)	30/232 (12.9)	1.86 (0.5–4.3)	0.14
Health care teamwork	4/7 (57.1)	32/250 (12.8)	4.46 (0.0–7.8)	< 0.01
Patient communication	2/5 (40.0)	34/252 (13.5)	2.96 (0.0–9.2)	0.12
Clinical assessment	32/51 (62.7)	4/206 (1.9)	32.31 (15.0–146.5)	< 0.01
COVID-19 related	18/61 (29.5)	18/196 (9.2)	3.21 (1.9–5.8)	< 0.01

^*^See Appendix Table 7 for groupings

**Table 4:** Adjusted Association Between Diagnostic Process Faults and Diagnostic Errors (*n* = 257)

DEER Diagnostic Process Fault^*^	Model 1—all covariates			Model 2—excluding clinical assessment		
	Adjusted PR (95% CI)	*p*	AF (95% CI)	Adjusted PR (95% CI)^†^	*p*	AF (95% CI)^†^
Access and presentation	0.75 (0.1 to 1.7)	0.48	− 3.09 (− 11.2 to 5.0)	0.81 (0.2 to 1.4)	0.59	− 3.12 (− 11.9 to 5.6)
History taking	1.33 (0.8 to 2.8)	0.18	13.18 (− 8.0 to 34.4)	2.15 (1.1 to 5.1)	< 0.01	28.77 (5.8 to 51.8)
Physical exam	1.66 (0.9 to 3.7)	0.06	11.58 (− 1.1 to 24.2)	2.95 (1.5 to 6.5)	< 0.01	20.16 (5.1 to 35.2)
Testing ordering, performance, and interpretation	1.55 (1.0 to 4.0)	0.08	18.53 (− 1.1 to 38.2)	3.21 (1.8 to 7.0)	< 0.01	36.72 (17.2 to 56.2)
Patient follow-up and monitoring	1.01 (0.3 to 2.2)	0.98	0.16 (− 12.4 to 12.7)	2.24 (1.1 to 5.1)	0.03	12.44 (− 2.5 to 27.3)
Consultation and referral	1.05 (0.1 to 2.1)	0.88	0.64 (− 8.5 to 9.8)	0.96 (0.3 to 2.4)	0.92	− 0.49 (− 12.0 to 11.0)
Health care teamwork	1.08 (0.1 to 6.5)	0.88	0.36 (− 6.3 to 7.0)	1.83 (0.0 to 7.1)	0.30	2.86 (− 6.0 to 11.7)
Patient communication	2.24 (0.0 to 7.8)	0.13	3.54 (− 2.7 to 9.7)	1.09 (0.0 to 6.1)	0.92	0.26 (− 4.6 to 5.1)
Clinical assessment	13.43 (4.8 to 81.4)	< 0.01	78.79 (55.6 to 102.0)	–	–	–
COVID-19 related	1.38 (0.8 to 2.7)	0.18	11.24 (− 5.1 to 27.6)	1.6 (0.8 to 2.9)	0.11	15.27 (− 3.8 to 34.4)

*PR* prevalence ratio, *AF* attributable fraction

^*^See Appendix Table 7 for groupings

^***†***^***Clinical assessment process fault grouping not included in the multivariable model***

**Table 5:** Diagnostic Errors in COVID-19 PUI Patients, March Through July 2020

Month	Case count by month (*n*, % overall)	Diagnostic error count by month (*n*, % of cases that month)
2/20 and 3/20^*^	80 (31.1)	8 (10.0)
4/20	52 (20.2)	6 (11.5)
5/20	47 (18.3)	5 (10.6)
6/20	44 (17.1)	11 (25.0)
7/20	34 (13.2)	6 (17.6)

^*^Includes 6 cases from February 2020; *p* value for trend = 0.06

**Table 6:** Harms Due to Diagnostic Errors

Potential severity of harm (*n* = 39)	*n*	(%)
Error did not reach the patient	6	(15.4)
Error reached the patient but did not cause harm	16	(41.0)
Error required monitoring to confirm that it resulted in no harm	3	(7.7)
Error that may have contributed to or resulted in temporary harm and required intervention	5	(12.8)
Error that may have contributed to or resulted in temporary harm and required initial or prolonged hospitalization	7	(17.9)
Error required intervention necessary to sustain life	1	(2.6)
Error that may have contributed to or resulted in the patient’s death	1	(2.6)

### Error Rates Over Time (Table 5)

Diagnostic error rates rose slightly in the last 2 months of the study period (June and July 2020), but this trend did not meet tests for statistical significance (*p* = 0.06).

### Harms Due to Diagnostic Errors (Table 6)

According to the NCC-MERP harms rating scale, 14 patients’ errors (35.9% of patients with errors) produced temporary or permanent harm or led to death; this estimate suggests that harmful errors were present in 5.4% of patients admitted with suspected COVID-19.

## DISCUSSION

In this multicenter retrospective study of patients admitted for evaluation for potential COVID-19 infection, diagnostic errors were common. Diagnostic errors were not associated with clinical features of or risk factors for COVID-19 infection or COVID-19 test turnaround times. In contrast, DEs were associated with more general diagnostic process faults such as problems with history taking, physical examination, test ordering, performance, or interpretation, and patient follow-up and monitoring; these factors were closely related to clinical assessment, which was the most common source of DEs. Interestingly, diagnostic process faults related to COVID-19 itself (such need for isolation or medical distancing) were not independently associated with DEs when broader causes of diagnostic process faults were considered.

Our diagnostic error rate needs to be interpreted in light of a field using varying definitions of errors and approaches to detecting cases for review. Studies using common morbid events or “triggers” (e.g., myocardial infarction or epidural abscesses) to identify patients where a diagnosis might have been missed estimated broad ranges of potential error rates (between 2 and 62%).^28^ Autopsy-based studies have suggested that missed diagnoses are present in 6% of autopsies^29^ while reviews of over- or under-diagnosis in unselected geriatric patients estimated an error rate of 10% or more.^30^ When DEs are evaluated as a subset of inpatient adverse events, lower estimated rates (0.2–2.7%) are seen.^31^ Our estimated rates fall well within this very broad range from previous studies, few of which used a structured review process to identify diagnostic errors or selected patients without an obvious adverse event to “trigger” consideration of an error. Thus, our findings likely represent a more generalized diagnostic error rate.

Patients with and without DEs were nearly identical in terms of patient factors such as age, comorbidities, language, and social determinants of health. Though language limitations and some social determinants of health were likely underdetected with chart reviews alone, it is unlikely that detection bias would have differentially affected patients with and without errors. Detection bias likely reduced our ability to find subtle associations between patient-level factors and diagnostic process faults. While the early phases of the pandemic struck older and disadvantaged patients most severely, our data do not suggest gaps in care or worsened outcomes extended to risks for DEs in vulnerable patient populations. Similarly, DEs were not associated with comorbidities or symptoms which might have been confused with COVID-19 infection (e.g., congestive heart failure or chronic lung diseases, which might also produce dyspnea and infiltrates on chest radiography). Similarly, recognized features of COVID-19 disease risk factors (such as travel to high-risk areas or living in a congregate setting) did not appear to be associated with missed or delayed diagnoses of either COVID-19 or other illnesses. Although scarcity of COVID-19 testing and long test turnaround times were important barriers to timely diagnosis early in the pandemic, we did not find either factor to be associated with diagnostic errors.

In contrast, in multivariable models accounting for DEER process fault groupings, diagnostic errors were associated with general diagnostic process fault groupings, but not with faults specific to the COVID-19 pandemic. It is possible that diagnostic error propagation during the pandemic was driven less by changes caused by COVID-19, and more by foundational problems in diagnostic processes present in everyday care, such as failure to recognize deterioration or loss of information due to multiple handoffs in care. Although we do not have direct measures of workload or hospital capacity, these sorts of stressors, particularly during the beginning of the pandemic, may have had broad impact on a range of diagnostic processes by limiting physicians’ ability to spend adequate time with patients or do the cognitive work required to make accurate and timely diagnoses. In this vein, it is notable that delirium and social isolation were the only patient factors associated with DE, i.e., patients who may require more time spent to gather diagnostic information. Although we used broad DEER classifications as a framework for COVID-19-related faults, it is important to note that our adjudication training asked for reviewers to consider and classify COVID-19-related processes as explicitly separate concepts from standard clinical decision-making. Despite this structured approach, it is possible that our chart review process did not detect subtle issues such as anchoring on COVID-19 diagnoses or communication gaps, making these factors both less common and less associated with DEs after adjusting for more general fault types. Finally, it is also possible that stresses of the pandemic, particularly use of PPE and high workload, may have made physicians less likely to document details of patient history or their diagnostic thinking, which would in turn reduce our ability to discern associations with diagnostic errors. As mentioned above, it is unlikely that documentation biases would have differentially affected patients with or without errors, but this would have made it less likely we would have been able to detect items only gatherable via physician notes.

For similar reasons, we cannot disentangle the relationship between the most common fault associated with diagnostic errors—clinical assessment—and other faults related to history taking, physical exam, or diagnostic testing. For example, it is possible that testing and history taking process faults might lead to problems in clinical assessment (a step which depends on integration of other diagnostic processes). Alternatively, our data could point to the central role of clinical assessment as a root cause of other process faults; failure to consider a diagnosis in the first place can lead to errors in eliciting a piece of history, physical exam finding, or ordering a diagnostic test.

Our study has several limitations. We used chart reviews to gather data, which might be subject to documentation biases and detection biases. To overcome documentation biases, we encouraged chart reviewers to use all available documentation in the medical record (e.g., discharge summaries, admission notes, progress notes, nursing notes, test results, orders, etc.) and to use a reasonable judgment framework to interpret notes and ordering patterns. However, some aspects of care—such as communication between team members or between teams, patients, and families—may not have been captured in documentation and are likely underrepresented in our data. Our data also cannot directly measure whether a diagnostic error represented one type of cognitive process or another (for example, the provider did not consider COVID-19 was the leading diagnosis, but it was, vs. thinking it was COVID-19 but it was not). To address detection biases, all reviewers underwent extensive training at study outset, and cases were overread and reviewed by members of the core research team to ensure consistency and validity. Because PUI admissions varied across our hospitals and over time, our sampling strategy likely resulted in a different proportion of overall PUI admissions being reviewed at each site. While this limits sample size and power within sites, our randomization approach and rigorous and stringent exclusion processes mitigate selection and detection biases. It is possible that local reviewers’ adjudications were shaped by local norms and professional standards (e.g., expectations for consultation timeliness). We addressed this potential problem via training and ongoing inter-site over-read of cases. Our study was not able to capture information about larger challenges in health care at the time of our study, for example, hospital census or physician workload. Finally, although our study incorporated data from multiple hospitals, the overall sample size in our cohort was relatively small and may limit generalizability and statistical power of our findings.

In summary, this multicenter study of diagnostic errors among patients admitted with consideration of COVID-19 as a potential diagnosis demonstrated that diagnostic errors were relatively common and not associated with symptoms, signs, or risk factors associated with COVID-19 or with care processes put in place in the early phase of the pandemic. Several of the process areas associated with diagnostic errors — such as test ordering, history taking, or physical examination gaps — may represent target areas for educational and quality improvement efforts and may be particularly vulnerable to periods of stress in the health care system.

## Data Availability

The data that support the findings of this study are available from the corresponding author upon reasonable request.

## Appendix 1

Table 7

**Table 7:** Frequencies of individual DEER process faults (n = 257)

DEER Taxonomy Process Fault	N (%)
**Access and Presentation Faults**	
Failure or delay in seeking care due to COVID-19^*^	14 (5.4)
Failure or delay in seeking care unrelated to COVID-19	14 (5.4)
Preadmission care provided remotely^*^	15 (5.8)
Failure or denial of access to care	7 (2.7)
Failure of triage or admission to wrong service	6 (2.3)
Delay of care in the ED	1 (0.4)
Delay in transfer from outside hospital	4 (1.6)
Inability to obtain needed care	1 (0.4)
**History Taking Faults**	
Failure or delay in providing a critical piece of history data due to less communication with patient or family due to COVID-19^*^	6 (2.3)
Failure or delay in providing a critical piece of history data due to less communication with patient or family unrelated to COVID-19	13 (5.1)
Inaccurate or misinterpreted piece of data	6 (2.3)
Suboptimal weighing of a piece of data	18 (7.0)
Failure or delaying acting on or following up on a piece of history data	7 (2.7)
Over-reliance on second-hand history information	6 (2.3)
Patient/caregiver unable to provide history	36 (14.0)
Failure or delay in accessing data from EHR	4 (1.6)
**Physical Exam Faults**	
Failure or delay in eliciting critical physical exam finding due to COVID-19^*^	17 (6.6)
Failure or delay in eliciting critical physical exam finding unrelated to COVID-19	4 (1.6)
Inaccurate or misinterpreted physical exam finding	6 (2.3)
Suboptimal weighing of a physical exam finding	16 (6.2)
Failure or delay in acting on or following up on a physical exam finding	2 (0.8)
**Test Ordering, Performance, and Interpretation Faults**	
Failure or delay in ordering needed tests	24 (9.3)
Failure to order correct tests	1 (0.4)
Failure or delay in performing needed tests	10 (3.9)
Suboptimal test sequencing	3 (1.2)
Failure to order tests in correct way	0
Identification failure-mislabeled specimen	0
Technical or processing error	2 (0.8)
Specimen delivery problem	0
Erroneous reading of test by lab	6 (2.3)
Failure or delay of reporting test results to clinician	3 (1.2)
Erroneous clinician interpretation of test result related to COVID-19^*^	5 (1.9)
Erroneous interpretation of test unrelated to COVID-19	9 (3.5)
**Patient Follow-up and Monitoring Faults**	
Failure or delay in acting on or following up on test result	10 (3.9)
Failure or delay in monitoring	0
Missed physiologic monitoring finding	8 (3.1)
Failure or delay in recognizing or acting upon urgent condition or complications due to medical distancing for COVID-19^*^	3 (1.2)
Failure or delay in recognizing or acting upon urgent condition or complications unrelated to COVID-19	2 (0.8)
Failure to refer patient to appropriate setting or for appropriate monitoring	3 (1.2)
Failure or delay in timely re-examination of the patient	1 (0.4)
**Consultation/Referral Faults**	
Failure or delay in consultation due to COVID-19^*^	3 (1.2)
Failure or delay in ordering a referral or consult unrelated to COVID-19	5 (1.9)
Failure or delay of the consulting team to see the patient	1 (0.4)
Suboptimal consultation performance	6 (2.3)
Use of virtual visit instead of in-person consult^*^	14 (5.4)
**Healthcare Team Communication and Collaboration Faults**	
Failure or delay in communication of information between primary team and pathologists, radiologists, or technologists	1 (0.4)
Failure or delay in communication between the consultants and the primary team	1 (0.4)
Failure or delay in communication of information within the care team due to fragmentation of the care team due to COVID-19^*^	0
Failure or delay in communication of information within the care team due to fragmentation of the care team unrelated to COVID-19	2 (0.8)
Failure or delay in communication among consultants with each other	1 (0.4)
Inadequate oversight or supervision of trainees or advanced practice provider	2 (0.8)
**Patient Experience Faults**	
Failure to communicate an accurate and timely explanation of the patient’s health problems to the caregiver	2 (0.8)
Failure or delay in communicating lab/test results, assessment, or consultant findings to the caregiver	0
Failure to identify or address patient or caregiver concerns, preferences, or non-adherence	3 (1.2)
**Clinical Assessment Faults**	
Failure or delay in considering the diagnosis	36 (14.0)
Suboptimal weighing or prioritizing	29 (11.3)
Failure or delay in recognizing complications	11 (4.3)
Anchoring on COVID-19 as the diagnosis^*^	20 (7.8)

^*^Added to capture diagnostic process faults related to COVID-19 care
